# Effects of Heat Adaptation Behaviors on Resting Heart Rate Response to Summer Temperatures in Older Adults: Wearable Device Panel Study

**DOI:** 10.2196/67721

**Published:** 2025-11-14

**Authors:** Chi-Hsien Chen, Feipei Lai, Yu-Lin Chen, Yue Leon Guo

**Affiliations:** 1 Department of Environmental and Occupational Medicine, National Taiwan University College of Medicine and National Taiwan University Hospital, Rm 339, 17 Syujhou Road, Taipei, 100, Taiwan, 886 2 3322 821, 886 2 3322 8214; 2 Department of Computer Science and Information Engineering, National Taiwan University, Taipei, Taiwan; 3 School of Environmental and Forest Sciences, University of Washington, Seattle, Washington, United States; 4 Institute of Environmental and Occupational Health Sciences, National Taiwan University, Taipei, Taiwan; 5 National Institute of Environmental Sciences, National Health Research Institutes, Zhunan Township, Miaoli County, Taiwan

**Keywords:** temperature, heat, adaptation behavior, resting heart rate, wearable device, modification effect, mobile phone

## Abstract

**Background:**

The health impact of summer heat on older adults is a growing public concern, yet the physiological responses, particularly changes in resting heart rate (RHR), and the role of personal heat adaptation behaviors remain underexplored. Wearable devices offer an opportunity to objectively monitor physiological responses and evaluate the effectiveness of adaptation strategies in real-world settings.

**Objective:**

This study aimed to quantify the short-term association between summer temperatures and RHR in older adults and to examine how individual heat adaptation behaviors modify this relationship, with additional consideration of personal characteristics such as age, sex, BMI, and chronic disease status.

**Methods:**

We conducted a panel study among 83 community-dwelling older adults (≥65 y) in Taipei City during the summer of 2021 (May to September). Participants wore Garmin smartwatches to continuously monitor heart rate. Daily RHR was defined as the lowest 30-minute average heart rate. In September, heat adaptation behaviors were assessed via structured telephone interviews. Ambient temperature and relative humidity were obtained from a nearby monitoring station. Linear mixed-effect models were used to estimate temperature-RHR associations, and interaction terms were included to examine behavioral modifications. Subgroup analyses were conducted to explore effect modification by individual characteristics such as age, sex, BMI, and chronic disease status.

**Results:**

Each 1 °C increase in daily mean temperature over lag days 0‐1 was associated with a 0.11 (95% CI 0.07-0.15; *P*<.001) beats/min increase in RHR. After mutual adjustment for behaviors, several heat adaptation strategies showed significant protective effects, including reducing physical activity (*β*=−.15, *P*=.001), drinking cold beverages (*β*=−.24, *P*<.001), increasing naps or sleep duration (*β*=−.28, *P*=.003), drinking additional water ≥500 mL (*β*=−.10, *P*=.02), using air conditioner (AC) before (*β*=−.15, *P*=.002) and during sleep (*β*=−.13, *P*=.007), and using electric fans during sleep (*β*=−.12, *P*=.01). Subgroup analyses revealed stronger effects for certain behaviors in vulnerable populations: reduced physical activity was particularly beneficial for those with higher BMI; AC use and cold beverage intake were more effective in people with diabetes; increased naps yielded the largest benefits in individuals with hypertension; and the use of AC or fans during sleep was especially protective for older adults and females.

**Conclusions:**

Summer heat is associated with elevated RHR in older adults, but this effect can be mitigated through targeted heat adaptation behaviors. Smartwatch monitoring provides a feasible and informative approach for capturing physiological changes, supporting the development of personalized heat-health recommendations for aging populations in a warming climate.

## Introduction

Many epidemiological studies have demonstrated the impact of extreme ambient heat exposure on all-cause and cardiovascular mortality [1-3]. The specific causes of cardiovascular mortality and morbidity include heart failure, stroke, ischemic heart disease, hypertensive heart disease, arrhythmias, and cardiac arrest [4,5]. In the context of global warming, the risk of heat-related cardiovascular mortality is increasing [6,7]. Older adult individuals are more susceptible to heat-related deaths [5], likely due to impaired cardiopulmonary reserve and thermoregulatory capability, as well as relative socioeconomic disadvantages [8,9].

On an individual level, identifying personal risk factors and implementing appropriate adaptive behaviors may help mitigate heat-related risks [8]. However, existing studies on the effectiveness of heat adaptation behaviors have notable limitations, including their reliance on self-reported symptoms, cross-sectional designs that limit causal inference, and inconsistent findings regarding the effectiveness of specific adaptation measures. For example, a study in Guangdong, China, demonstrated an association between drinking more water and wearing light clothing with a reduced risk of heatstroke [10]. In contrast, other studies have shown that the implementation of heat adaptation behaviors is linked to an elevated risk of heat-related illness or symptoms [11-14]. These inconsistencies highlight the need for more robust, longitudinal studies.

Most current studies depend on self-reported symptoms and behaviors, introducing potential reporting bias and reverse causality issues where individuals experiencing symptoms may be more likely to adopt adaptation behaviors. Our previous research has addressed some of these limitations by using effect modification analysis to demonstrate the benefits of heat adaptation behaviors in reducing heat-related symptoms associated with obesity [14]. Given that symptom reporting is inherently subjective, using objective physiological indicators remains critical to comprehensively assess the health impacts of heat exposure and validate the benefits of heat adaptation behaviors.

Heart rate plays a central role in thermoregulatory response and is affected by autonomic nerve and neurohormone regulations [15]. Resting heart rate (RHR) has been known as a significant predictor of mortality and morbidity [15]. According to a meta-analysis of 87 prospective studies, an increase of 10 beats per minute in baseline RHR is linked to a 15% rise in cardiovascular mortality and a 17% rise in all-cause mortality [16]. This suggests that even a single beat per minute increase in RHR is not clinically negligible.

However, RHR measurements can vary significantly under different conditions (eg, higher during the daytime than at nighttime and higher in a hospital setting than at home), which limits its clinical application and interpersonal comparison [17]. This challenge has been addressed by the increasing prevalence of wearable devices. Wearable devices using photoplethysmography sensors to measure heart rate usually provide good precision, especially in resting conditions [18-20]. Nevertheless, these devices present several limitations in real-world applications, including motion artifacts during physical activity, reduced signal accuracy in individuals with darker skin pigmentation or tattoos, variability caused by inconsistent wrist positioning and contact pressure, interference from ambient light and low temperatures, as well as interdevice variability in measurement precision [21-23]. Despite these limitations, wearable technology continues to serve as a valuable tool for objective, long-term ambulatory heart rate monitoring in nonclinical settings.

The objectives of this study are twofold—first, to investigate the impact of summer temperatures on the RHR of older adult individuals using wearable devices and time-series data analysis; and second, to evaluate the benefits of personal heat adaptation behaviors by examining the effect modification of heart rate. By integrating physiological data from wearable technology with behavioral adaptation patterns, this research aims to provide evidence-based recommendations for personal heat adaptation strategies. The findings can inform public health interventions targeting heat-vulnerable populations, particularly older adults, and support the development of personalized guidance to reduce heat-related cardiovascular risks in aging societies under climate change.

## Methods

### Study Design and Participants

This investigation was devised as a prospective, longitudinal cohort study aimed at examining the older adult population residing in the Taipei Basin, a region in northern Taiwan characterized by a humid subtropical climate (Köppen climate classification: Cfa). Between 1987 and 2009, Taipei City noted an average temperature increase of 1.57 °C per century, surpassing the global average warming rate of 0.6 °C per century, as indicated in the National Oceanic and Atmospheric Administration’s 2023 Annual Climate Report [24,25]. The Taipei Basin, which includes the densely populated cities of Taipei and New Taipei, is marked by a significant urban heat island effect due to its extensive urbanization. The Surface Urban Heat Island Index in Taipei during the summer reaches as high as 9 °C during the day and 3 °C at night, the highest among all cities in Taiwan [26].

Participants were recruited from an established older adult cohort, originally assembled between 2016 and 2018, to investigate the health impacts of air pollution [27]. The initial cohort was recruited from hospitals across 5 regions in Taiwan, where older adults attended government-funded health examinations. For this study, further enrollment took place between May and November 2020 [28]. Eligible participants were selected from the original cohort based on the following criteria: residence in the Taipei Basin, ownership of a smartphone compatible with the Garmin app, and the ability and willingness to wear a smartwatch consistently. Individuals with severe language, cognitive, or communication impairments were excluded. While leveraging an existing cohort provided a robust foundation, the requirement for smartphone ownership and wearable device usage may have introduced selection bias toward participants with higher socioeconomic status or greater technological proficiency, potentially limiting generalizability. However, the sample reflects a growing segment of older adults who are increasingly adopting mobile health technologies. Of the 97 cohort members eligible for this study, 14 were lost to follow-up due to refusal to continuously wear the device, resulting in 83 participants who provided sufficient wearable data for analysis. The study size was determined by the number of eligible and consenting participants; no formal sample size calculation was performed due to the exploratory panel design, which used repeated daily measurements to enhance statistical power. From September to October 2021, the participants were interviewed via telephone to gather information on their personal background, health condition, and behaviors related to heat adaptation. To explore the impact of summer temperatures on heart rate, the study extracted heart rate data from May to September 2021 for statistical analysis.

### Ethical Considerations

The study was conducted in accordance with the principles outlined in the Declaration of Helsinki and received approval from the Institutional Review Board of National Taiwan University Hospital (IRB 202003049RINB). All participants were recruited voluntarily and provided written informed consent before their participation. They were informed of their right to withdraw from the study at any time without consequence. No financial compensation was provided for participation. All collected data were deidentified before analysis to ensure participant privacy and confidentiality.

### Questionnaire Assessment

A structured questionnaire was developed to gather data on various aspects, including personal habits (such as cigarette smoking and alcohol consumption), comorbidities (like diagnosed diabetes mellitus, hypertension, and heart diseases), educational levels, and adaptation strategies for dealing with heat and its related symptoms. Telephone interviews were conducted by an experienced interviewer who had previously administered face-to-face interviews with the participating older adult individuals using the same questionnaire [14]. A specific question, “What was the method from the following list that you used to adapt to heat during the extremely hot days in the past year?” was used to identify 15 adaptation behaviors.

For behaviors involving the use of air conditioners (ACs) or electric fans, participants were asked to report whether they used these devices during specific time periods of the day (morning, noon, afternoon, before sleep, and during sleep) and to indicate usage if it typically totaled more than 1 hour within each period, including intermittent use.

As the data relied on self-reported behaviors, particularly over a 2‐4 month recall period, responses may have been subject to recall bias. Evidence from recall error studies indicates that a 90-day recall period yields the lowest bias, compared with the tendency for overreporting with shorter recall periods (eg, 30 d) and underreporting with longer periods (eg, 365 d) [29]. To help mitigate this, interviews were conducted by a familiar interviewer using a standardized protocol to improve consistency and participant comfort. In future studies, the incorporation of real-time behavior logging through mobile apps may further improve data accuracy and reduce recall-related limitations.

### RHR Measurement

Study participants were directed to wear a Garmin Vivosmart 4 smartwatch (Garmin International Inc) on their preferred wrist for continuous monitoring of their heart rate. At the initiation of the wearable device setup, participants’ height and weight were measured. The device uses photoplethysmography sensors, which detect changes in blood volume by measuring variations in light absorption through the skin. The heart rate data were uploaded to the Garmin Connect platform via a smartphone-internet connection and then retrieved from the National Taiwan University Medical Genie Platform [30], integrated via the Garmin Health application programming interface. Participants were instructed to wear the smartwatch consistently throughout the day and night, ensuring a minimum daily usage of 8 hours, with the daily RHR determined from the lowest 30-minute average within a 24-hour span [31].

In older adult individuals, the Garmin Vivosmart series has shown good accuracy for RHR monitoring, with a concordance correlation coefficient of 0.93 compared with electrocardiogram measurements from the Polar H7 chest-strapped heart rate monitor [32]. However, despite its strengths, photoplethysmography-based heart rate monitoring may be affected by age-related skin changes—such as wrinkles, roughness, pigmentation, dilated blood vessels, and reduced skin tone—which may alter light absorption and degrade signal quality [33,34]. In addition, vascular aging and increased arterial stiffness in older adults may influence pulse waveforms and reduce measurement precision [35]. These limitations should be considered when interpreting heart rate data in older populations.

### Exposure Assessment

In this study, the hourly values of air temperature and relative humidity were obtained from the Environmental Protection Agency air pollution monitoring stations located closest to the participants’ residences. The monitoring stations, positioned 13 to 15 meters above ground level, used Metone 083R instruments to measure temperature and humidity with accuracies of 0.1 °C and ±1% at 23 °C, respectively. The distance between each participant’s residence and the nearest monitoring station ranged from 0.23 to 4.29 kilometers, with a mean distance of 1.74 kilometers. For analysis, air temperature and relative humidity measurements were averaged on a daily basis. The heat index was calculated using these daily average values based on the algorithm released by the US National Weather Service in 2011 [36], with the detailed algorithm and equation provided in Method S1 in Multimedia Appendix 1.

### Statistical Analysis

A linear mixed-effect (LME) model was used to evaluate the association between personal characteristics, meteorological conditions, and RHR, incorporating random intercepts for individual participants to account for repeated measurements within participants. For the assessment of the impact of temperature and humidity on RHR, the LME model included adjustments for age (continuous variable), sex (male or female), hypertension (yes or no), diabetes (yes or no), heart diseases (yes or no), and educational attainment (<13 or ≥13 y). Days with missing RHR data (11,016/12,699, 13.3%) due to nonwear were excluded from the analysis.

To assess the modifying effects of personal characteristics and adaptive behaviors, subgroup analysis was performed based on age (≤ or > median), sex (female or male), hypertension (no or yes), diabetes (no or yes), heart disease (no or yes), BMI (≤ or > median), educational attainment (<13 or ≥13 y), and each adaptive behavior (no or yes). The *P* value for the interaction term in the LME model was used to evaluate the statistical significance of effect modification.

To better evaluate the independent impact of each adaptive behavior on heart rate response to heat, we performed 2 mutually adjusted models for various heat adaptation behaviors. In the first model, AC and electric fan use across different time periods were mutually adjusted to identify which specific usage patterns had significant modifying effects. In the second model, the significant AC and electric fan usage behaviors identified in the first model were further adjusted alongside other adaptive behaviors that had shown significant modifying effects when tested individually.

Furthermore, a 3-way interaction term was applied in the LME model to examine whether the modifying effect of heat adaptation behaviors depends on personal characteristics.

To assess the robustness of the association between ambient temperature and RHR, we conducted a series of sensitivity analyses using progressively adjusted models. Model 1 was unadjusted (crude); model 2 adjusted for age, sex, and BMI; model 3 additionally included hypertension, diabetes, and heart disease; and model 4 (the final fully adjusted model) further accounted for educational attainment. All models were estimated using LME models with random intercepts for individuals. In addition, the aforementioned subgroup analyses were used to examine whether specific demographic or clinical subgroups exhibited differential RHR responses to heat exposure, further supporting the consistency and robustness of the observed associations. All analyses were executed using JMP 17 (JMP Statistical Discovery LLC), with all statistical tests being 2-sided and a *P* value of <.05 deemed statistically significant.

## Results

### Participant Characteristics

In this study, 83 older adult participants wore wearable devices throughout the warm season from May to October 2021, averaging 132.7 days of use and providing 11,016 RHR measurements. Details on their demographics, duration of device use, and heat adaptation methods are outlined in Table 1. The participants, with an average age of 73.7 (SD 3.6) years, had chronic disease rates of 15% (12/83) for diabetes, 42% (35/83) for hypertension, and 15% (12/83) for heart disease. These rates are slightly lower than previously reported national estimates among Taiwanese older adults, where the prevalence of hypertension and diabetes has been found to range from 48% to 60% and 20% to 24%, respectively [37,38]. This may suggest that our sample represents a relatively healthier segment of the older adult population, possibly because participants were initially recruited from individuals who attended older adult health check-up programs, reflecting greater health awareness and engagement.

They reported an average of 8.4 different heat adaptation behaviors, with the most prevalent including wearing light clothes (79/83, 95%), using AC (78/83, 94%) and electric fans (77/83, 93%), and using shade strategies such as umbrellas or hats (67/83, 81%), and staying indoors (60/83, 72%). Drinking more water (58/83, 70%) and increasing bathing or showering frequency (50/83, 60%), along with opening windows (49/83), were also common practices.

**Table 1.** Distribution of characteristics and wearable device indicators among 83 participants.

Characteristics and wearable device indicators	Value
Age (y)	
Mean (SD)	73.7 (3.6)
Median (IQR)	73.3 (70.1-75.0)
Sex (male), n (%)	39 (40)
Hypertension, n (%)	35 (42)
Diabetes, n (%)	12 (15)
Heart diseases, n (%)	12 (15)
BMI	
Mean (SD)	23.7 (2.5)
Median (IQR)	23.4 (21.6-25.6)
Education <13 years, n (%)	16 (19)
Adaptation behaviors for heat	
Use electric fans, n (%)	77 (93)
Morning	49 (59)
Noon	70 (84)
Afternoon	70 (84)
Night before sleep	70 (84)
Night during sleep	59 (71)
Wear light cloth, n (%)	79 (95)
Drink more water (extra ≥500 mL), n (%)	58 (70)
Use air conditioner, n (%)	78 (94)
Morning	7 (8)
Noon	37 (45)
Afternoon	35 (42)
Night before sleep	38 (46)
Night during sleep	56 (68)
Use public air conditioner, n (%)	22 (27)
Days per week using air conditioner	
Mean (SD)	5.6 (2.1)
Median (IQR)	7 (5-7)
Use umbrella or hat, n (%)	67 (81)
Stay in the shade, n (%)	61 (74)
Open window, n (%)	49 (59)
Increase frequency of bathing or shower, n (%)	50 (60)
Heated water for showering, n (%)	47 (57)
Stay indoors, n (%)	60 (72)
Reduce physical activity, n (%)	34 (41)
Drink cold water or beverage, n (%)	18 (22)
Eat more fruit, n (%)	35 (42)
Increase time for nap or sleep, n (%)	5 (6)
Number of adaptive behaviors	
Mean (SD)	8.4 (1.6)
Median (IQR)	8 (7-10)
Duration of wearable device (d)	
Mean (SD)	132.7 (36.9)
Median (IQR)	153 (132-153)
Daily resting heart rate (beats/min)	
Mean (SD)	58.4 (7.1)
Median (IQR)	59 (54-63)

### Device Adherence and Missing Data

The number of participants providing RHR values each day is illustrated in Figure S1 in Multimedia Appendix 1. A gradual decline in device wear over the study period (from 83 to 63 participants) suggests potential adherence fatigue or reduced engagement. Missing RHR data were primarily due to smartwatch nonwear, which occurred more frequently on hotter days, likely due to discomfort or device removal during heat exposure (Figure S2 in Multimedia Appendix 1). Missingness was not associated with age (Figure S3 in Multimedia Appendix 1), but this pattern may have resulted in a mildly underrepresentation of high-temperature days.

### Environmental Conditions

**Figure 1.**
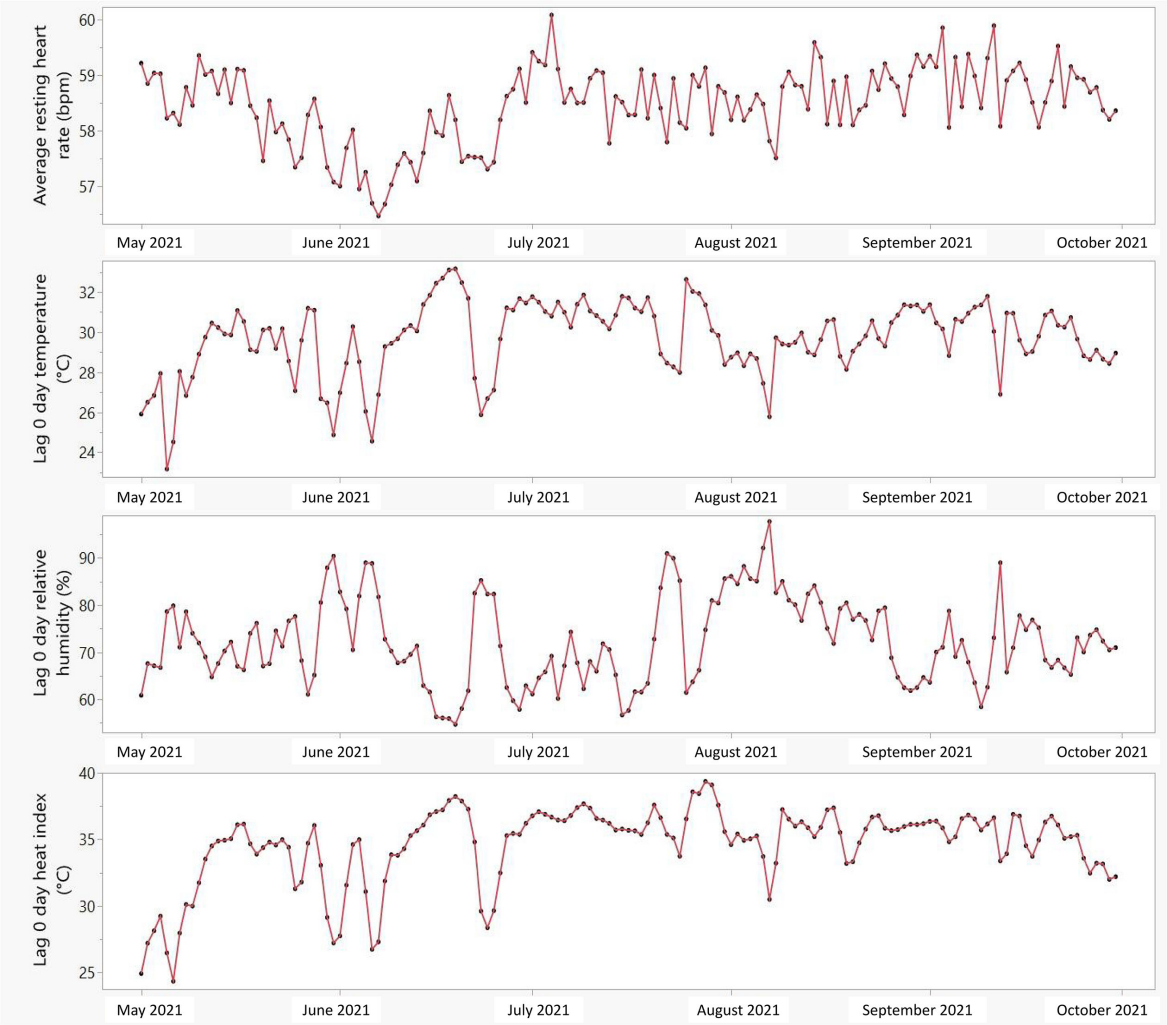
Daily resting heart rate, temperature, humidity, and heat index during the study period.

The distribution of temperature and humidity during the study period is summarized in Multimedia Appendix 2 and illustrated in Figure 1. The average temperature was 29.6 °C (SD 1.9 °C), with a mean humidity of 72.1% (SD 9.3%), and the heat index averaged 34.2 °C (SD 3.1 °C). Temperature and humidity were strongly inversely correlated (Spearman ρ=−0.82, *P*<.001), while temperature and heat index were positively correlated (ρ=0.84, *P*<.001).

### Associations Between Individual Characteristics and RHR

Multimedia Appendix 3 outlines the results from univariate regression analyses linking individual traits with RHR. Diabetes was associated with a higher RHR, with a regression coefficient of 3.95 (95% CI 0.29‐7.60; *P*=.04), while hypertension and heart diseases did not show significant effects. No significant correlations were found with age, sex, BMI, or education level. The morning use of electric fans initially correlated with higher heart rates (coefficient=2.76, 95% CI 0.14‐5.37; *P*=.04), but this association became nonsignificant after adjusting for personal characteristics (data not shown). Other heat adaptation behaviors did not significantly affect RHR.

### Associations Between Temperature and RHR

Table 2 shows that temperature and the heat index positively correlate with RHR on the same and following day, with effects diminishing and turning negative by Lag 4 and 5. Humidity negatively correlates with RHR initially, with significance lasting through Lag 2, but becomes nonsignificant thereafter. For example, each 1.9 °C increase in ambient temperature (1 SD) was associated with a 0.19 (95% CI 0.12-0.26; *P*<.001) beats per minute increase in RHR on Lag 0 . Model fit statistics favored ambient temperature over heat index as the exposure metric: both Corrected Akaike Information Criterion and Bayesian Information Criterion were consistently smaller for temperature-based models, supporting its use as the primary indicator of heat exposure in this study.

**Table 2.** Association between temperature and humidity and resting heart rate in various lag days. Coefficients were estimated for an SD increase in temperature (1.9 ℃), relative humidity (9.3%), and heat index (3.1 ℃) by mixed effect models with random intercept for individuals and with adjustments for age, sex, BMI, hypertension, diabetes, heart diseases, and education attainment.

Condition and lag day	Coefficient (95% CI)	*P* value
Air temperature		
Lag 0 day	0.19 (0.12 to 0.26)	<.001
Lag 1 day	0.19 (0.11 to 0.26)	<.001
Lag 2 day	0.08 (0.01 to 0.15)	.03
Lag 3 day	−0.03 (−0.10 to 0.04)	.43
Lag 4 day	−0.09 (−0.16 to −0.02)	.02
Lag 5 day	−0.09 (−0.16 to −0.02)	.02
Lag 6 day	−0.06 (−0.13 to 0.02)	.13
Lag 7 day	−0.07 (−0.14 to 0.01)	.08
Relative humidity		
Lag 0 day	−0.21 (−0.28 to −0.13)	<.001
Lag 1 day	−0.23 (−0.30 to −0.16)	<.001
Lag 2 day	−0.17 (−0.24 to −0.1)	<.001
Lag 3 day	−0.06 (−0.13 to 0.02)	.13
Lag 4 day	−0.003 (−0.08 to 0.07)	.94
Lag 5 day	−0.03 (−0.1 to 0.05)	.49
Lag 6 day	−0.05 (−0.12 to 0.03)	.21
Lag 7 day	−0.04 (−0.12 to 0.03)	.25
Heat index		
Lag 0 day	0.19 (0.12 to 0.27)	<.001
Lag 1 day	0.18 (0.11 to 0.25)	<.001
Lag 2 day	0.06 (−0.01 to 0.14)	.09
Lag 3 day	−0.03 (−0.11 to 0.04)	.41
Lag 4 day	−0.08 (−0.16 to −0.01)	.03
Lag 5 day	−0.08 (−0.16 to −0.01)	.03
Lag 6 day	−0.05 (−0.13 to 0.02)	.18
Lag 7 day	−0.06 (−0.14 to 0.01)	.09

### Sensitivity Analyses

Sensitivity analyses using progressively adjusted models (Table S1 in Multimedia Appendix 1) demonstrated that the association between ambient temperature and RHR was consistent across all models, from the unadjusted model to the fully adjusted model, including age, sex, BMI, comorbidities, and educational attainment. This indicates that the observed associations are robust and not substantially influenced by confounding from the included covariates.

### Effect Modification by Personal Characteristics and Heat Adaptation Behaviors

Subgroup analyses demonstrated that the association between ambient temperature and RHR varied significantly by personal characteristics and heat adaptation behaviors (Table 3). Sex modified the response, with males showing a greater RHR increase per 1 °C rise in temperature (coefficient=0.20, 95% CI 0.14‐0.27) than females (coefficient=0.06, 95% CI 0.003‐0.11; for interaction *P*<.001). A similar pattern was seen for BMI, where those above the median had stronger responses than those below (0.13 vs 0.10; for interaction *P*=.02). Participants without diabetes had a significant RHR increase (0.14 beats per minute [bpm]), while those with diabetes showed no clear change (–0.09 bpm; for interaction *P*<.001). Lower education levels were also associated with stronger RHR responses.

**Table 3.** Effect modification of personal conditions and adaptation behaviors on the relationship of lag 0‐1 day temperature and resting heart rate. Subgroup analyses were done by mixed effect models with random intercept for individuals and with adjustments for age, sex, BMI, hypertension, diabetes, heart diseases, and education. Coefficients were estimated for 1℃ increase in temperature.

Conditions and adaptation behaviors	Coefficient (95% CI)	*P* value	*P* for interaction
Age (y)			
≤Median	0.10 (0.04 to 0.15)	<.001	.42^a^
>Median	0.13 (0.07 to 0.19)	<.001	.16^b^
Sex			
Male	0.20 (0.14 to 0.27)	<.001	<.001
Female	0.06 (0.003 to 0.11)	.04	—^c^
Hypertension			
No	0.12 (0.06 to 0.17)	<.001	.75
Yes	0.10 (0.05 to 0.16)	<.001	—
Diabetes			
No	0.14 (0.1 to to 0.19)	<.001	<.001
Yes	−0.09 (−0.2 to 0.03)	.15	—
Heart diseases			
No	0.10 (0.05 to 0.14)	<.001	.14
Yes	0.19 (0.09 to 0.28)	<.001	—
BMI			
≤Median	0.10 (0.04 to 0.15)	<.001	.48^a^
>Median	0.13 (0.07 to 0.19)	<.001	.02^b^
Education <13 y			
No	0.14 (0.09 to 0.18)	<.001	.02
Yes	0.01 (−0.08 to 0.1)	.80	—
Adaptation behaviors for heat			
Use electric fans			
No	0.28 (0.10 to 0.46)	.002	.03
Yes	0.10 (0.06 to 0.14)	<.001	—
Morning			
No	0.10 (0.04 to 0.16)	<.001	.77
Yes	0.12 (0.06 to 0.17)	<.001	—
Noon			
No	0.09 (−0.02 to 0.19)	.10	.63
Yes	0.12 (0.07 to 0.16)	<.001	—
Afternoon			
No	0.07 (−0.04 to 0.19)	.20	.45
Yes	0.12 (0.08 to 0.16)	<.001	—
Night before sleep			
No	0.08 (−0.02 to 0.19)	.13	.55
Yes	0.12 (0.07 to 0.16)	<.001	—
Night during sleep			
No	0.20 (0.13 to 0.26)	<.001	.008
Yes	0.08 (0.03 to 0.13)	.003	—
Wear light cloth			
No	0.19 (0.03 to 0.34)	.02	.39
Yes	0.11 (0.07 to 0.15)	<.001	—
Drink more water (extra ≥500 mL)			
No	0.24 (0.17 to 0.30)	<.001	<.001
Yes	0.05 (0.00 to 0.11)	.04	—
Use air conditioner			
No	0.33 (0.14 to 0.52)	<.001	.01
Yes	0.10 (0.06 to 0.14)	<.001	—
Morning			
No	0.13 (0.08 to 0.17)	<.001	.03
Yes	−0.03 (−0.15 to 0.09)	.59	—
Noon			
No	0.17 (0.11 to 0.22)	<.001	<.001
Yes	0.05 (−0.01 to 0.11)	.98	—
Afternoon			
No	0.15 (0.09 to 0.20)	<.001	.06
Yes	0.07 (0.01 to 0.13)	.03	—
Night before sleep			
No	0.20 (0.14 to 0.26)	<.001	<.001
Yes	0.01 (−0.05 to 0.06)	.84	—
Night during sleep			
No	0.19 (0.11 to 0.26)	<.001	.01
Yes	0.07 (0.03 to 0.12)	.002	—
Use public air conditioner			
No	0.16 (0.11 to 0.21)	<.001	<.001
Yes	−0.02 (−0.10 to 0.06)	.61	—
Days per week using air conditioner			
0-6 d	0.22 (0.15 to 0.28)	<.001	<.001^a^
7 d	0.04 (−0.01 to 0.09)	.14	<.001^b^
Use umbrella or hat			
No	0.03 (−0.06 to 0.12)	.48	.07
Yes	0.13 (0.08 to 0.18)	<.001	—
Stay in the shade			
No	0.06 (−0.04 to 0.16)	.22	.17
Yes	0.13 (0.09 to 0.17)	<.001	—
Open window			
No	−0.004 (−0.07 to 0.06)	.90	<.001
Yes	0.19 (0.14 to 0.24)	<.001	—
Increase frequency of bathing or shower			
No	0.13 (0.06 to 0.19)	<.001	.58
Yes	0.10 (0.05 to 0.16)	.0002	—
Heated water for showering			
No	0.07 (0.00 to 0.14)	.04	.09
Yes	0.14 (0.09 to 0.19)	<.001	—
Stay indoors			
No	0.09 (0.01 to 0.16)	.02	.48
Yes	0.12 (0.07 to 0.17)	<.001	—
Reduce physical activity			
No	0.16 (0.11 to 0.22)	<.001	.005
Yes	0.04 (−0.02 to 0.11)	.15	—
Drink cold water or beverage			
No	0.16 (0.12 to 0.21)	<.001	<.001
Yes	−0.09 (−0.19 to 0.05)	.06	—
Eat more fruit			
No	0.16 (0.10 to 0.22)	<.001	.01
Yes	0.05 (−0.01 to 0.10)	.08	—
Increase time for nap or sleep			
No	0.13 (0.08 to 0.17)	<.001	.005
Yes	−0.12 (−0.27 to 0.02)	.09	—
Number of adaptive behaviors			
<8	0.21 (0.14 to 0.28)	<.001	<.001^a^
≥8	0.06 (0.01 to 0.11)	.03	<.001^b^

a Represented categorical variables in interaction terms.

b Represented continuous variables in interaction terms.

c Not available.

Several adaptation behaviors were linked to reduced RHR increases. Using AC (0.07 vs 0.19 bpm; *P*=.01), electric fans during sleep (0.08 vs 0.20 bpm; for interaction *P*=.008), and drinking extra water (0.05 vs 0.24 bpm; for interaction *P*<.001) were protective. Other beneficial behaviors included drinking cold beverages (−0.09 vs 0.16 bpm; for interaction *P*<.001), eating more fruit (0.05 vs 0.16 bpm; for interaction *P*=.01), and increasing sleep or nap time (−0.12 vs 0.13 bpm; for interaction *P*=.005). In contrast, opening windows was associated with greater RHR increases (0.19 bpm; *P*<.001). Notably, participants engaging in 8 or more adaptation behaviors had significantly lower RHR responses compared with those with fewer behaviors (0.06 vs 0.21 bpm; for interaction *P*<.001), suggesting a cumulative protective effect. These results highlight the modifying roles of individual health factors and behavioral adaptations in shaping physiological responses to heat.

### Multivariate Models of Heat Adaptation Behaviors

To assess the independent effects of specific heat adaptation behaviors, we conducted 2 mutually adjusted models evaluating their interaction with temperature on RHR (Table 4). Model m1 examined the effect of AC and electric fan use across different times of day. Among these, using AC before sleep (coefficient=−0.19, 95% CI –0.29 to −0.08; *P*<.001) and during sleep (coefficient=−0.15, 95% CI −0.24 to −0.05; *P*=.002) were significantly associated with attenuated RHR responses to temperature. Electric fan use during sleep also demonstrated a protective effect (coefficient=−0.19, 95% CI −0.31 to −0.07; *P*=.002). Fan use during other time periods and daytime AC use did not show statistically significant effects.

**Table 4.** Regression coefficients of interaction terms with mutual adjustment of various heat adaptation behaviors to assess modification effects. Coefficients denote the regression coefficients of interaction terms between each adaptation behavior and temperature on resting heart rate. Coefficients were estimated for 1℃ increase in temperature.

Adaptation behaviors	Coefficient (95% CI)	*P* value
Model m1: mutual adjustment of electric fan and AC^a^ usage patterns over various time periods		
Use AC in the morning	−0.12 (−0.3 to 0.06)	.18
Use AC at noon	−0.03 (−0.13 to 0.06)	.50
Use AC in the afternoon	−0.04 (−0.14 to 0.06)	.41
Use AC before sleep	−0.19 (−0.29 to −0.08)	<.001
Use AC during sleep	−0.15 (−0.24 to −0.05)	.002
Use electric fans in the morning	0.03 (−0.07 to 0.13)	.57
Use electric fans at noon	0.02 (−0.18 to 0.23)	.83
Use electric fans in the afternoon	0.19 (−0.06 to 0.45)	.14
Use electric fans before sleep	−0.16 (−0.4 to 0.08)	.19
Use electric fans during sleep	−0.19 (−0.31 to −0.07)	.002
Model m2: mutual adjustment for significant adaptation behaviors		
Open window	0.05 (−0.06 to 0.15)	.39
Reduce physical activity	−0.15 (−0.23 to −0.06)	.001
Drink cold water or beverage	−0.24 (−0.35 to −0.13)	<.001
Increase naps or sleep	−0.28 (−0.46 to −0.1)	.003
Drink more water (extra ≥500 mL)	−0.10 (−0.19 to −0.01)	.02
Eat more fruit	−0.06 (−0.15 to 0.02)	.13
Use AC before sleep	−0.15 (−0.24 to −0.05)	.002
Use AC during sleep	−0.13 (−0.22 to −0.03)	.007
Use electric fans during sleep	−0.12 (−0.22 to −0.03)	.01

a AC: air conditioner.

Model m2 included multiple significant behaviors from previous subgroup analyses, allowing for mutual adjustment. The most protective effects were observed for increasing nap or sleep time (−0.28, 95% CI −0.46 to −0.10; *P*=.003), and drinking cold beverages (−0.24, 95% CI −0.35 to −0.13; *P*<.001). Other behaviors with significant effects included reducing physical activity (−0.15, 95% CI −0.23 to −0.06; *P*=.001), drinking extra water (−0.10, 95% CI −0.19 to −0.01; *P*=.02), and continued support for AC use before or during sleep and fan use during sleep.

In contrast, opening windows and eating more fruit did not significantly modify RHR response to temperature after adjustment for other behaviors. These findings highlight that certain behaviors, particularly those that directly cool the body or reduce exertion, have independent and additive effects in mitigating physiological stress during heat exposure.

### Effect Modification of Heat Adaptation Behaviors by Participant Characteristics

Subgroup analyses revealed that the effectiveness of heat adaptation behaviors in mitigating RHR response to temperature varied significantly across individual characteristics (Table 5). Opening windows was associated with a greater increase in RHR among individuals with higher BMI compared with those with lower BMI (0.30 vs 0.10 bpm, for interaction *P*=.02), suggesting that this behavior may expose more heat-vulnerable individuals to excessive ambient temperatures.

Reducing physical activity was more protective in participants with heart disease (−0.33 vs −0.08 bpm, for interaction *P*=.03) and those with higher BMI (−0.28 vs 0.03 bpm, for interaction *P*<.001), indicating that limiting exertion may be particularly beneficial for those with cardiometabolic risks. Similarly, drinking cold beverages led to a stronger reduction in RHR among older adults (−0.41 vs −0.14 bpm, for interaction *P*=.02), males (−0.39 vs −0.16 bpm, for interaction *P*=.048), and individuals with diabetes (−0.46 vs −0.12 bpm, for interaction *P*=.007).

**Table 5.** Subgroup analysis by individual characteristics for the modification effects of several adaptation behaviors. Coefficients denote the regression coefficients of interaction terms between each adaptation behavior and temperature on resting heart rate. Coefficients were estimated for 1℃ increase in temperature.

Characteristics	Coefficient (95% CI)	*P* for interaction^a^
Open window		
Age (y), <median	0.21 (0.10 to 0.32)	.64
Age (y), ≥median	0.17 (0.05 to 0.29)	—^b^
Sex, male	0.25 (0.12 to 0.38)	.29
Sex, female	0.16 (0.05 to 0.26)	—
HTN^c^, no	0.13 (0.01 to 0.25)	.08
HTN, yes	0.28 (0.17 to 0.39)	—
DM^d^, no	0.17 (0.08 to 0.26)	.34
DM, yes	0.28 (0.05 to 0.52)	—
Heart disease, no	0.21 (0.12 to 0.30)	.11
Heart disease, yes	−0.02 (−0.26 to 0.22)	—
BMI (kg/m^2^), <median	0.10 (−0.02 to 0.22)	.02
BMI (kg/m^2^), ≥median	0.30 (0.19 to 0.42)	—
Reduce physical activity		
Age (y), <median	−0.05 (−0.16 to 0.06)	.13
Age (y), ≥median	−0.18 (−0.30 to −0.06)	—
Sex, male	−0.18 (−0.31 to −0.05)	.17
Sex, female	−0.06 (−0.17 to 0.05)	—
HTN, no	−0.19 (−0.31 to −0.07)	.047
HTN, yes	−0.02 (−0.13 to 0.09)	—
DM, no	−0.11 (−0.20 to −0.03)	.71
DM, yes	−0.16 (−0.40 to 0.08)	—
Heart disease, no	−0.08 (−0.17 to 0.01)	.03
Heart disease, yes	−0.33 (−0.52 to −0.14)	—
BMI (kg/m^2^), <median	0.03 (−0.08 to 0.14)	<.001
BMI (kg/m^2^), ≥median	−0.28 (−0.41 to −0.16)	—
Drink cold water or beverage		
Age (y), <median	−0.14 (−0.27 to −0.02)	.02
Age (y), ≥median	−0.41 (−0.58 to −0.24)	—
Sex, male	−0.39 (−0.56 to −0.23)	.04
Sex, female	−0.16 (−0.29 to −0.04)	—
HTN, no	−0.32 (−0.46 to −0.18)	.12
HTN, yes	−0.16 (−0.30 to −0.01)	—
DM, no	−0.12 (−0.24 to 0.01)	.007
DM, yes	−0.46 (−0.70 to −0.23)	—
Heart disease, no	−0.25 (−0.35 to −0.14)	—
Heart disease, yes	—	—
BMI (kg/m^2^), <median	−0.35 (−0.49 to −0.21)	.06
BMI (kg/m^2^), ≥median	−0.15 (−0.30 to 0.00)	—
Increase naps or sleep		
Age (y), <median	−0.17 (−0.42 to 0.09)	.39
Age (y), ≥median	−0.32 (−0.55 to −0.08)	—
Sex, male	0.03 (−0.24 to 0.29)	.01
Sex, female	−0.43 (−0.65 to −0.20)	—
HTN, no	−0.05 (−0.26 to 0.16)	<.001
HTN, yes	−1.06 (−1.40 to −0.72)	—
DM, no	−0.29 (−0.46 to −0.12)	—
DM, yes	—	—
Heart disease, no	−0.37 (−0.58 to −0.17)	.03
Heart disease, yes	0.07 (−0.23 to 0.37)	—
BMI (kg/m^2^), <median	−0.32 (−0.51 to −0.13)	.08
BMI (kg/m^2^), ≥median	0.07 (−0.33 to 0.48)	—
Drink more water (extra ≥500 mL)		
Age (y), <median	−0.26 (−0.39 to −0.12)	.15
Age (y), ≥median	−0.13 (−0.25 to −0.01)	—
Sex, male	−0.09 (−0.24 to 0.05)	.09
Sex, female	−0.25 (−0.36 to −0.14)	—
HTN, no	−0.23 (−0.35 to −0.11)	.17
HTN, yes	−0.10 (−0.24 to 0.03)	—
DM, no	−0.14 (−0.23 to −0.05)	.08
DM, yes	−0.48 (−0.88 to −0.08)	—
Heart disease, no	−0.23 (−0.33 to −0.14)	.007
Heart disease, yes	0.10 (−0.09 to 0.29)	—
BMI (kg/m^2^), <median	−0.31 (−0.43 to −0.20)	.002
BMI (kg/m^2^), ≥median	−0.03 (−0.17 to 0.12)	—
Eat more fruit		
Age (y), <median	−0.18 (−0.29 to −0.07)	.06
Age (y), ≥median	−0.02 (−0.14 to 0.10)	—
Sex, male	−0.09 (−0.22 to 0.04)	.84
Sex, female	−0.10 (−0.21 to 0.00)	—
HTN, no	−0.13 (−0.24 to −0.01)	.64
HTN, yes	−0.09 (−0.20 to 0.03)	—
DM, no	−0.17 (−0.26 to −0.09)	<.001
DM, yes	0.26 (0.02 to 0.50)	—
Heart disease, no	−0.11 (−0.20 to −0.02)	.62
Heart disease, yes	−0.05 (−0.25 to 0.14)	—
BMI (kg/m^2^), <median	−0.04 (−0.15 to 0.07)	.10
BMI (kg/m^2^), ≥median	−0.18 (−0.31 to −0.06)	—
Use AC before sleep		
Age (y), <median	−0.12 (−0.24 to −0.01)	.09
Age (y), ≥median	−0.27 (−0.39 to −0.15)	—
Sex, male	−0.27 (−0.4 to −0.14)	.15
Sex, female	−0.15 (−0.25 to −0.04)	—
HTN, no	−0.25 (−0.36 to −0.14)	.11
HTN, yes	−0.12 (−0.23 to 0.00)	—
DM, no	−0.13 (−0.21 to −0.04)	.009
DM, yes	−0.47 (−0.73 to −0.21)	—
Heart disease, no	−0.20 (−0.29 to −0.11)	.82
Heart disease, yes	−0.17 (−0.36 to 0.02)	—
BMI <median	−0.19 (−0.31 to −0.07)	.64
BMI (kg/m^2^), ≥median	−0.23 (−0.35 to −0.11)	—
Use AC during sleep		
Age (y), <median	−0.17 (−0.29 to −0.06)	.21
Age (y), ≥median	−0.06 (−0.19 to 0.07)	—
Sex, male	0.07 (−0.07 to 0.20)	.001
Sex, female	−0.22 (−0.34 to −0.11)	—
HTN, no	−0.14 (−0.26 to −0.02)	.44
HTN, yes	−0.07 (−0.19 to 0.04)	—
DM, no	−0.10 (−0.20 to −0.01)	.31
DM, yes	−0.23 (−0.47 to 0.01)	—
Heart disease, no	−0.10 (−0.20 to 0.00)	.86
Heart disease, yes	−0.12 (−0.31 to 0.06)	—
BMI (kg/m^2^), <median	−0.13 (−0.25 to −0.01)	.67
BMI (kg/m^2^), ≥median	−0.09 (−0.22 to 0.03)	—
Use electric fans during sleep		
Age (y), <median	0.09 (−0.03 to 0.22)	<.001
Age (y), ≥median	−0.30 (−0.42 to −0.17)	—
Sex, male	0.06 (−0.08 to 0.19)	.002
Sex, female	−0.23 (−0.35 to −0.11)	—
HTN, no	−0.25 (−0.37 to −0.12)	.002
HTN, yes	0.03 (−0.09 to 0.15)	—
DM, no	−0.08 (−0.17 to 0.01)	—
DM, yes	—	—
Heart disease, no	−0.13 (−0.24 to −0.03)	.335
Heart disease, yes	−0.02 (−0.20 to 0.17)	—
BMI (kg/m^2^), <median	−0.25 (−0.37 to −0.14)	.002
BMI (kg/m^2^), ≥median	0.04 (−0.10 to 0.19)	—

a The *P* value for the interaction refers to the effects of the 3-way interaction between personal characteristics, adaptation behaviors, and temperature on resting heart rate.

b Not applicable.

c HTN: hypertension.

d DM: diabetes mellitus.

Increasing nap or sleep time showed sex-specific and disease-related differences in effect. The behavior led to greater RHR reductions in females than males (−0.43 vs 0.03 bpm, for interaction *P*=.01), in participants with hypertension versus without hypertension (−1.06 vs −0.05 bpm, for interaction *P*<.001), and in those without heart disease compared with those with heart disease (−0.37 vs 0.07 bpm, for interaction *P*=.03). Drinking additional water (≥500 mL) was also more effective in participants with lower BMI (−0.31 vs −0.03 bpm, for interaction *P*=.002) and those without heart disease (−0.23 vs 0.10 bpm, for interaction *P*=.007).

Fruit consumption showed opposing effects between people with diabetes and without diabetes: while it was associated with a decrease in RHR among people without diabetes (−0.17 bpm), it was paradoxically linked to an increase among people with diabetes (0.26 bpm, for interaction *P*<.001). This finding suggests potential metabolic differences in behavioral responses.

The use of AC also showed heterogeneity. AC use before sleep was more protective among people with diabetes (–0.47 vs –0.13 bpm, *P*=.009), while AC use during sleep had a stronger effect in females than males (–0.22 vs 0.07 bpm, *P*=.001). Similarly, the use of electric fans during sleep was associated with a greater reduction in RHR among older adults (–0.30 vs 0.09 bpm, *P*<.001), females (–0.23 vs 0.06 bpm, *P*=.002), participants without hypertension (–0.25 vs 0.03 bpm, *P*=.002), and those with lower BMI (–0.25 vs 0.04 bpm, *P*=.002).

These results underscore that not all adaptation behaviors are equally effective across individuals. Tailoring behavioral recommendations based on age, sex, BMI, and chronic disease status may enhance the protective effects of personal strategies during heat exposure.

## Discussion

### Principal Findings and Personalized Heat Adaptation

The results of this longitudinal study highlight the significant influence of summer temperatures on the RHR of older adult individuals, with clear evidence demonstrating that heat adaptation behaviors can effectively mitigate these impacts. By leveraging wearable device technology, we captured objective physiological data, revealing lag and interaction patterns in heart rate responses to varying temperatures and humidity levels. Our findings underscore the importance of adaptive behaviors such as using AC, using electric fans during sleep, increasing water intake, reducing physical activity, increasing rest time, and drinking cold water, all of which were shown to have a protective effect on RHR. Notably, these adaptive strategies appeared to be more beneficial for certain subgroups: reducing physical activity for individuals with higher BMI, using electric fans during sleep and drinking cold water for the older age group, increasing rest time for participants with hypertension, and drinking cold water and using AC before sleep for participants with diabetes. However, eating more fruit may not be an ideal heat adaptation behavior for patients with diabetes. These results emphasize the need for personalized heat adaptation recommendations.

### Comparison With Previous Research and Mechanistic Insights

The observed RHR response to summer temperature (0.1 beats/min per 1 °C) was consistent with findings from a large cross-sectional study in China (0.133 beats/min per 1 °C) [39]. Heart rate can serve as an early indicator of heat effects, as the increase in heart rate occurs before the rise in core body temperature at the inflection point of heat response [40]. The physiological mechanism involves an increase in heart rate due to heat stimulating the sympathetic nervous system, while stroke volume is limited due to reduced venous return, as blood pools in the skin vessels to dissipate more heat [41,42]. Another follow-up study showed that temperature increases during the warm season decrease parasympathetic modulation of heart rate and modify baroreflex sensitivity in as little as 6 hours [43]. The immediate response to heat aligns with our findings of peak RHR response to heat on lag 0‐1 days.

### Interpretation of Humidity-Related Effects

In this study, humidity exhibited a negative correlation with both temperature and RHR. This phenomenon may be related to the frequent afternoon rainfall during summer in Taiwan. In addition to increasing humidity, the evaporation process during rainfall absorbs a significant amount of heat energy, leading to a decrease in air temperature [44]. Furthermore, the cloud cover before and after the rainfall blocks sunlight, reducing solar radiation reaching the ground and further lowering the temperature [45]. Solar radiation significantly influences human thermal sensation by elevating skin temperature and perceived heat load, thereby reducing self-regulated outdoor exercise intensity [46-48]. Although high humidity impairs the body’s ability to dissipate heat through sweat evaporation, thereby increasing heat strain and reducing self-paced exercise performance under hot conditions [49,50], the observed negative association between humidity and RHR may reflect its collinear relationship with concurrent reductions in temperature and solar radiation exposure.

### Behavioral Adaptation Versus Physiological Response

Previous research [10-13], including our own [14], has shown a significant positive correlation between personal heat adaptation behaviors and self-reported heat-related symptoms. However, in this study, no direct significant correlation was found between personal heat adaptation behaviors and RHR. This suggests that individuals adjust their behaviors in response to perceived heat-related symptoms but are unable to perceive cardiovascular impacts in advance to take preventive measures.

Through the analysis of effect modification, we can clearly demonstrate the impact of heat adaptation behaviors on heart rate response to heat. In our previous research on heat-related symptoms, significant effects were only observed when combining 2 adaptive behaviors [14]. However, for RHR, a single adaptive behavior alone has an independently significant modifying effect, demonstrating beneficial effects on physiological response.

### Effectiveness of AC, Especially at Night

The benefits of AC stem from maintaining a cooler, more stable indoor environment, thereby reducing thermal stress. Studies from various countries, including the United States [51,52], Spain [52,53], China [54], Canada [52], and Japan [52,55], have shown that AC use can lower heat-related mortality. A randomized controlled trial demonstrated that providing targeted heat health messages increased AC use and reduced the risk of self-reported heat stress among older adults [56]. Our study extends these findings by demonstrating the specific impact of AC use on an objective physiological measure—RHR—highlighting its potential role in preventing heat-related cardiovascular events. Furthermore, the stronger protective effect of AC use at night highlights the significance of nighttime heat, which aligns with previous literature showing the impact of nighttime warming on mortality, especially under the urban heat island effect [57]. For patients with diabetes, nighttime AC is even more beneficial in lowering RHR, likely due to their increased vulnerability to heat exposure caused by impaired thermoregulatory and autonomic nerve responses [58].

### Protective Role and Limitations of Electric Fan Use

Electric fans enhance convective and evaporative heat loss from the skin, significantly improving thermal comfort while consuming up to 50 times less electricity than AC [59]. However, at extremely high temperatures, such as around 36 °C with 80% relative humidity or 42 °C with 50% relative humidity, electric fans can exacerbate physiological heat strain [60]. In addition, the cooling efficacy diminishes with age and in conditions that impair sweating efficiency [61]. According to biophysiological modeling, the recommended safe temperature thresholds for electric fan use are 39 °C for healthy adults aged 18‐40 years, 38 °C for those older than 65 years, and 37 °C for older adults on anticholinergic medications [59]. During the study period, nighttime temperatures ranged between 28 °C and 32 °C (average 28 °C), which is below the recommended safe temperature thresholds for electric fan use. This may explain the observed protective effects of nighttime electric fan use for older adults.

The protective effect of electric fan use during sleep on RHR was more pronounced in senior participants and women, potentially because these groups are more susceptible to heat-related hazards and have reduced sudomotor function [5,62]. Conversely, no protective effect was observed in individuals with higher levels of obesity, likely due to subcutaneous fat reducing the surface area-to-mass ratio, limiting heat exchange with the environment [63]. Furthermore, sweat gland density is lower in areas with higher adipose content, weakening heat dissipation through sweat evaporation [64]. Similarly, individuals with hypertension did not show a protective effect of electric fan use on RHR during sleep, unlike people without hypertension. This may be attributed to heightened sympathetic nervous system activity in people with hypertension [65], leading to skin vasoconstriction and diminished water vapor evaporation from the skin [66]. In addition, certain antihypertensive medications, such as beta-blockers, calcium-channel blockers, and diuretics, reduce heart rate and cardiac contractility, while also increasing the risk of dehydration [67].

### Modifying Physical Activity and Sleep During Heat Exposure

Reducing physical activity lowers the RHR response to heat exposure because physical exertion increases metabolic heat production. Combined with environmental heat, this exacerbates heat stress [8]. The protective effect of reduced physical activity is likely more pronounced in individuals with heart disease and those with a higher BMI, as the added internal heat may increase strain on their cardiovascular system. In these populations, compromised cardiac function and an already elevated cardiac workload make them more vulnerable to heat stress [68].

In addition to the protective benefit of reducing physical activity during heatwaves, increasing naps or sleep also demonstrated independent protective effects, likely due to the enhancement of autonomic balance through increased parasympathetic activity and reduced sympathetic activity associated with longer daytime naps [69] and extended nighttime sleep [70]. The stronger effect observed in females may be linked to generally lower physical fitness levels and reduced thermoregulatory capacity [71], while the heightened response in individuals with hypertension could be attributed to elevated sympathetic nerve activity [65]. The observation that individuals with heart disease experienced less benefit from increased naps or sleep compared with those without heart disease may be explained by the high prevalence of sleep-disordered breathing in patients with heart conditions [72].

### Effects of Cold Water Consumption and Hydration

Our study found that drinking cold water significantly reduced the RHR response to heat, with a more pronounced effect observed in males, those with diabetes, and older adults. This aligns with previous research, including a randomized crossover study demonstrating that cold and room-temperature water increase vagal activity, leading to a greater reduction in heart rate compared with body-temperature water [73]. A systematic review further supports these findings, noting that cold beverages help attenuate core temperature and improve performance in the heat [74]. The stronger effects observed in our subgroups may be explained by physiological differences: men have been shown to experience greater core temperature increases after repeated exertion [75] and stronger parasympathetic activation during cooling tasks [76]. People with diabetes and older adults often exhibit impaired thermoregulation [8], heightened sympathetic activity, and reduced parasympathetic tone [77,78], which may amplify the benefits of cold water on RHR. Further research is necessary to clarify the mechanisms behind these effects and their broader implications for mitigating heat-related health risks.

In our mutually adjusted model, both increased water intake and cold water exhibited independent protective effects. Hydration replenished fluids, maintaining blood volume, cardiac preload, and output, thereby enhancing skin blood flow, facilitating heat dissipation, and promoting the restoration of heart rate variability [79,80]. However, participants with heart disease and higher BMI showed a blunted response to increased water consumption. In patients with heart disease, maintaining fluid balance can be challenging, and the ability to augment cardiac output is less efficient through fluid supplement, potentially due to the limitations imposed by the Frank-Starling mechanism [81]. Similarly, increased BMI is associated with impaired left ventricular diastolic function, which reduces the effectiveness of fluid supplementation on improving cardiac function [82].

### Reconsidering the Role of Window Opening

Opening windows during heatwaves was a common adaptation in our study, with 59% (49/83) of participants reporting this behavior. However, we found that opening windows increased the RHR response to heat. This finding aligns with our previous research, which showed that this behavior intensified the association between body fat and heat-related symptoms (not opening windows: odds ratio [OR] 0.91, 95% CI 0.07-11.12; opening windows: OR 1.71, 95% CI 1.06-1.29; interaction *P*=.19), suggesting a potentially harmful effect. On hot days, opening windows can increase the inflow of hot air, raising indoor temperatures more effectively than heat conducted through the building [14]. In the mutually adjusted model, the effect of window opening became nonsignificant, likely due to confounding effects from other adaptation behaviors, such as the tendency to close windows when using AC [83]. In summary, although opening windows is a commonly used adaptation during hot weather, it does not appear to reduce heat-related health risks.

### Fruit Consumption and Heat Response in Patients With Diabetes

In our study, the heat adaptation behavior of consuming more fruits among patients with diabetes was found to increase the RHR response to heat. A meta-analysis of 19 randomized controlled trials has shown that, without exceeding total energy intake, increased fresh and dried fruit consumption can aid in glycemic control [84]. However, whether the observed phenomenon in our study is due to excessive intake remains uncertain. A limitation of this study is the lack of information on individual total caloric intake, as well as whether the fruits were consumed as whole fruit or fruit juice. Taiwan’s summer is characterized by an abundance of high-sugar fruits such as watermelon, passion fruit, pineapple, mango, longan, and sugar apple. Given that increased fruit consumption is a common adaptation behavior (reported by 35/83, 42% of participants), further research is warranted to clarify the impact of this dietary behavior on heat-related health risks in patients.

### Strengths

This study has several strengths. First, continuous measurement of RHR via a smartwatch provides an objective assessment of physiological responses to heat. Second, compared with studies focusing on subjective heat-related symptoms [14], integrating RHR with adaptation behaviors minimizes the risk of reverse causality, as individuals generally do not consciously perceive their heart rate response to heat, which might otherwise influence their behavior. Third, the use of moderation and mutually adjusted statistical analysis strategies allowed us to delineate the independent health benefits of different heat adaptation behaviors.

### Limitations

Several limitations should be acknowledged. First, the relatively small sample size of 83 participants, all from the Taipei Metropolitan Area, may limit the generalizability of our findings to the broader older adult population, particularly those in different geographic regions or climates. However, our study may serve as a model for assessing the health impacts of summer heat and developing region-specific personal adaptation strategies. Second, the collection of heat adaptation behavior data through telephone interviews conducted in September introduces the potential for recall bias, as participants were asked to recall behaviors from the preceding months. This reliance on self-reported data may also lead to inaccuracies in reporting. Third, the cross-sectional nature of the adaptation behavior data prevents the assessment of changes in behaviors over time or in response to specific heat events. Fourth, smartwatch nonwear was more common on hotter days, possibly due to discomfort or device removal during heat exposure. This pattern of missingness may lead to underrepresentation of high-temperature days in the dataset and result in conservative estimates of the heat’s effect on RHR. In addition, using meteorological data from monitoring stations may introduce some exposure misclassification, as it does not account for indoor conditions and outdoor activities that affect individual exposure. Wearable thermosensors could offer a future solution [18]. Nonetheless, such misclassifications are likely nondifferential and may result in an underestimation of the observed associations.

### Conclusion

By integrating objective physiological monitoring with self-reported behavioral data, this study provides a novel contribution to the field of environmental health by moving beyond subjective symptom reporting to evaluate the real-time cardiovascular effects of heat and the modifying role of personal adaptation strategies. This combination enables more accurate assessments of heat stress responses and intervention effectiveness in older adult populations.

Our findings demonstrate that common heat adaptation behaviors can significantly reduce cardiovascular strain during hot weather, particularly in older adults. Based on subgroup-specific responses, several behaviors should be prioritized for health promotion: using AC (especially before and during sleep), drinking cold water or beverages, reducing physical activity during hot periods, and increasing rest or nap time. These targeted strategies may be especially effective for vulnerable subgroups, including individuals with diabetes, hypertension, higher BMI, or advanced age.

This information may guide public health education and risk communication campaigns aimed at older adult populations, particularly in regions experiencing intensified heat due to climate change. Future research using enhanced time-series tracking and wearable technologies may further refine personalized adaptation guidance and support climate-resilient health systems.

## Supplementary material

10.2196/67721 Multimedia Appendix 1 Heat index formula; distribution of weather conditions; univariate regression coefficients to resting heart rate; sensitivity analysis; and participation rate by age, time, and temperature.

10.2196/67721 Multimedia Appendix 2 Distribution of temperature and humidity during the study period.

10.2196/67721 Multimedia Appendix 3 Univariate regression coefficients to resting heart rate.

## Abbreviations:

AC: air conditioner
bpm: beats per minute
LME: linear mixed-effect
OR: odds ratio
RHR: resting heart rate
