# Supplementary material for: Effects of Heat Adaptation Behaviors on Resting Heart Rate Response to Summer Temperatures in Older Adults: Wearable Device Panel Study
Source: JMIR Mhealth Uhealth. 2025 Nov 14;13:e67721. doi: 10.2196/67721 (PMC12617830; doi:10.2196/67721)
Supplement: Multimedia Appendix 1 [file mhealth-v13-e67721-s001.docx]

**Method S1**

Heat Index Equation Algorithm of the U.S. National Weather Service (NWS) in 2011

Step 1: Check for Cold Temperatures

If the ambient temperature (T) is 40°F or below, then:

Heat Index (HI) = T

Step 2: Preliminary Estimate for Mild Conditions

If T > 40°F, compute an estimate:

A = –10.3 + 1.1 × T + 0.047 × H

where H is the relative humidity (%).

If A < 79°F, then:

HI = A

Step 3: Heat Index Calculation for Warmer Conditions

If A ≥ 79°F, compute the heat index:

B = -42.379 + 2.04901523 × T + 10.14333127 × H
 - 0.22475541 × T × H - 0.00683783 × T²
 - 0.05481717 × H² + 0.00122874 × T² × H
 + 0.00085282 × T × H² - 0.00000199 × T² × H²

Step 4: Adjustment for Extremely Low or High Humidity

If H ≤ 13% and T is between 80°F and 112°F, adjust B:

HI = B - ((13 − H)/4) × √[(17 − |T − 95|)/17]

If H ≥ 85% and T is between 80°F and 87°F, adjust B:

HI = B + 0.02 × (H − 85) × (87 − T)

If no adjustment conditions apply, then:

HI = B

**Figure S1.**

Daily number of participants with resting heart rate records during the study period (May 2021 to September 2021)

**Figure S2.**

Relationship between ambient temperature and the number of participants providing resting heart rate data per day.

Beta=-0.81, SE=0.26, *p*=.002, R^2^=0.06

**Figure S3.**

Relationship between age and the number of days with valid resting heart rate data per participant.

Beta=0.71, SE=1.15, *p*=.541, R^2^=0.005

**Table S1.**

Sensitivity analyses of the association between ambient temperature and resting heart rate across lag days under varying levels of covariate adjustment.

|  | Coefficient | 95% CI | *p* value |  |  |
| --- | --- | --- | --- | --- | --- |
| Model 1: unadjusted | |  |  |  |  |
| Lag 0 day | 0.19 | (0.12,0.26) | <.0001 |  |  |
| Lag 1 day | 0.19 | (0.11,0.26) | <.0001 |  |  |
| Lag 2 day | 0.08 | (0.01,0.15) | 0.031 |  |  |
| Lag 3 day | -0.03 | (-0.10,0.04) | 0.437 |  |  |
| Lag 4 day | -0.09 | (-0.16,-0.02) | 0.016 |  |  |
| Lag 5 day | -0.09 | (-0.16,-0.02) | 0.016 |  |  |
| Lag 6 day | -0.06 | (-0.13,0.02) | 0.136 |  |  |
| Lag 7 day | -0.06 | (-0.14,0.01) | 0.083 |  |  |
| Model 2: adjusted for age, sex, and body mass index | | | | | |
| Lag 0 day | 0.19 | (0.12,0.26) | <.0001 |  |  |
| Lag 1 day | 0.19 | (0.11,0.26) | <.0001 |  |  |
| Lag 2 day | 0.08 | (0.01,0.15) | 0.031 |  |  |
| Lag 3 day | -0.03 | (-0.10,0.04) | 0.436 |  |  |
| Lag 4 day | -0.09 | (-0.16,-0.02) | 0.016 |  |  |
| Lag 5 day | -0.09 | (-0.16,-0.02) | 0.016 |  |  |
| Lag 6 day | -0.06 | (-0.13,0.02) | 0.135 |  |  |
| Lag 7 day | -0.07 | (-0.14,0.01) | 0.083 |  |  |
| Model 3: adjusted for age, sex, body mass index, hypertension, diabetes, and heart disease | | | | | |
| Lag 0 day | 0.19 | (0.12,0.26) | <.0001 |  |  |
| Lag 1 day | 0.19 | (0.11,0.26) | <.0001 |  |  |
| Lag 2 day | 0.08 | (0.01,0.15) | 0.031 |  |  |
| Lag 3 day | -0.03 | (-0.10,0.04) | 0.434 |  |  |
| Lag 4 day | -0.09 | (-0.16,-0.02) | 0.016 |  |  |
| Lag 5 day | -0.09 | (-0.16,-0.02) | 0.016 |  |  |
| Lag 6 day | -0.06 | (-0.13,0.02) | 0.135 |  |  |
| Lag 7 day | -0.07 | (-0.14,0.01) | 0.083 |  |  |

Coefficients represent the estimated change in resting heart rate (beats per minute) per one standard deviation increase in temperature (1.9°C), based on linear mixed-effect models with random intercepts for individuals. Models were sequentially adjusted for potential confounders: Model 1 is unadjusted; Model 2 adjusts for age, sex, and body mass index; Model 3 further adjusts for hypertension, diabetes, and heart disease.
