# Supplementary material for: Effects of Heat Adaptation Behaviors on Resting Heart Rate Response to Summer Temperatures in Older Adults: Wearable Device Panel Study
Source: JMIR Mhealth Uhealth. 2025 Nov 14;13:e67721. doi: 10.2196/67721 (PMC12617830; doi:10.2196/67721)
Supplement: Multimedia Appendix 2 [file mhealth-v13-e67721-s002.docx]

| Environmental variables | Mean (SD) | Median (IQR^a^) |
| --- | --- | --- |
| Temperature (℃) | 29.6 (1.9) | 29.9 (28.6-31) |
| Humidity (%) | 72.1 (9.3) | 71 (65.5-78.8) |
| Heat index (℃) | 34.2 (3.1) | 35 (32.8-36.3) |

^a^25th-75th percentile.
