# Supplementary material for: Effects of Heat Adaptation Behaviors on Resting Heart Rate Response to Summer Temperatures in Older Adults: Wearable Device Panel Study
Source: JMIR Mhealth Uhealth. 2025 Nov 14;13:e67721. doi: 10.2196/67721 (PMC12617830; doi:10.2196/67721)
Supplement: Multimedia Appendix 3 [file mhealth-v13-e67721-s003.docx]

| Individual and behavioral characteristics | | | Coefficient (95% CI) | P value |
| --- | --- | --- | --- | --- |
| Age (y) | | | 0.13 (−0.24 to 0.50) | .49 |
| Sex (female or male) | | | −0.33 (−3.04 to 2.38) | .81 |
| Hypertension (yes or no) | | | 2.15 (−0.48 to 4.78) | .11 |
| Diabetes (yes or no) | | | 3.95 (0.29 to 7.60) | .04 |
| Heart diseases (yes or no) | | | −2.49 (−6.20 to 1.22) | .19 |
| BMI | | | 0.02 (−0.51 to 0.55) | .93 |
| Education <13 years (yes or no) | | | 0.27 (−3.08 to 3.62) | .87 |
| Adaptation behaviors for heat | | |  |  |
|  | Use electric fans (yes or no) | | 0.17 (−4.93 to 5.27) | .95 |
|  |  | Morning (yes or no) | 2.76 (0.14 to 5.37) | .04 |
|  |  | Noon (yes or no) | −0.96 (−4.59 to 2.67) | .60 |
|  |  | Afternoon (yes or no) | −1.75 (−5.36 to 1.86) | .34 |
|  |  | Night before sleep (yes or no) | −1.59 (−5.20 to 2.03) | .39 |
|  |  | Night during sleep (yes or no) | 1.95 (−0.93 to 4.83) | .18 |
|  | Wear light cloth (yes or no) | | −3.19 (−9.31 to 2.93) | .30 |
|  | Drink more water (extra ≥500 mL; yes or no) | | 1.47 (−1.39 to 4.33) | .31 |
|  | Use air conditioner (yes or no) | | 1.25 (−4.29 to 6.80) | .65 |
|  |  | Morning (yes or no) | 0.46 (−4.29 to 5.21) | .85 |
|  |  | Noon (yes or no) | −0.35 (−3.00 to 2.31) | .80 |
|  |  | Afternoon (yes or no) | −0.2 (−2.87 to 2.48) | .88 |
|  |  | Night before sleep (yes or no) | 2.06 (−0.55 to 4.68) | .12 |
|  |  | Night during sleep (yes or no) | −0.95 (−3.76 to 1.86) | .50 |
|  | Use public air conditioning (yes or no) | | −1.73 (−4.69 to 1.24) | .25 |
|  | Days per week using air conditioning (yes or no) | | −0.04 (−0.68 to 0.59) | .89 |
|  | Use umbrella or hat (yes or no) | | −2.01 (−5.33 to 1.31) | .23 |
|  | Stay in the shade (yes or no) | | −1.23 (−4.21 to 1.75) | .41 |
|  | Open window (yes or no) | | 1.07 (−1.60 to 3.75) | .43 |
|  | Increase frequency of bathing or shower (yes or no) | | −0.86 (−3.55 to 1.83) | .53 |
|  | Heated water for showering (yes or no) | | 0.71 (−1.95 to 3.37) | .60 |
|  | Stay indoors (yes or no) | | 0.25 (−2.70 to 3.20) | .87 |
|  | Reduce physical activity (yes or no) | | −0.05 (−2.73 to 2.64) | .97 |
|  | Drink cold water or beverage (yes or no) | | 0.44 (−2.77 to 3.64) | .79 |
|  | Eat more fruit (yes or no) | | −1.08 (−3.74 to 1.59) | .42 |
|  | Increase time for nap or sleep (yes or no) | | −2.47 (−7.99 to 3.06) | .38 |
| Number of adaptive behaviors | | | −0.34 (−1.16 to 0.48) | .41 |
